# Secondhand smoking, knowledge/attitudes and socioeconomic status among married Bangladeshi women: a cross-sectional study

**DOI:** 10.1590/1516-3180.2018.0292071218

**Published:** 2019-05-08

**Authors:** Mosiur Rahman, Sheikh Mohammad Mahmudul Hasan, Syed Emdadul Haque, Nuruzzaman Haque, Mosfequr Rahman, Golam Mostofa, Sarwar Zahan, Durrul Huda, Saber Al- Sobaihi, Kapil Ahmed, Howlader Mohammad Miraz Mahmud

**Affiliations:** I MSc, MHSc, PhD. Associate Professor, Department of Population Science and Human Resource Development, University of Rajshahi, Rajshahi, Bangladesh, and JSPS Postdoc Fellow, Department of Global Health Entrepreneurship, Division of Public Health, Tokyo Medical and Dental University, Tokyo, Japan.; II; III; IV PhD. Professor, Department of Population Science and Human Resource Development, University of Rajshahi, Rajshahi, Bangladesh.; V PhD. Associate Professor, Department of Population Science and Human Resource Development, University of Rajshahi, Rajshahi, Bangladesh.; VI PhD. Professor, Department of Population Science and Human Resource Development, University of Rajshahi, Rajshahi, Bangladesh.; VII PhD. Senior Educational Professional, Institute of Educational Development, BRAC University, Dhaka, Bangladesh.; VIII MBBS. Director, Diabetic Association, Chapai Nawabganj, Bangladesh.; IX PhD. Postdoc fellow, Department of Global Health Entrepreneurship, Division of Public Health, Tokyo Medical and Dental University, Tokyo, Japan.; X MPH, PhD. Deputy Director (Research and Evaluation), Bangladesh Center for Communication Programs (BCCP), Dhaka, Bangladesh.; XI MSc. Research Coordinator (Research and Evaluation), Bangladesh Center for Communication Programs (BCCP), Dhaka, Bangladesh.

**Keywords:** Tobacco smoke pollution, Social class, Knowledge, Attitude, Bangladesh

## Abstract

**BACKGROUND:**

There is a paucity of research on knowledge/attitudes regarding the dangers of exposure to secondhand smoking (SHS) among women. The relationship between exposure to SHS, socioeconomic status (SES) and knowledge/attitudes regarding the risks of SHS has often been ignored. We therefore aimed to examine (1) whether SES and exposure to SHS were independently associated with knowledge/attitudes regarding the risks of SHS; and (2) whether women with low SES and exposure to SHS were uniquely disadvantaged in terms of deficient knowledge and more dismissive attitudes towards the risks of SHS.

**DESIGN AND SETTING:**

Cross-sectional study in the Rajshahi district, Bangladesh.

**METHODS:**

A total of 541 women were interviewed. Knowledge of and attitudes towards the risks of SHS were the outcomes of interest.

**RESULTS:**

A majority of the respondents were exposed to SHS at home (49.0%). Only 20.1% had higher levels of knowledge, and only 37.3% had non-dismissive attitudes towards the risks of SHS. Participants in the low SES group and those exposed to SHS had lower odds of higher knowledge and their attitudes towards the risks of SHS were more dismissive. Regarding deficient levels of knowledge and scores indicating more dismissive attitudes, women in the low SES group and who were exposed to SHS were not uniquely disadvantaged.

**CONCLUSIONS:**

Exposure to SHS and low SES were independently associated with deficient knowledge and scores indicating more dismissive attitudes. Regarding knowledge/attitudes, the negative effect of exposure to SHS extended across all socioeconomic backgrounds and was not limited to women in either the low or the high SES group.

## INTRODUCTION

Health promotion programs worldwide have long been premised on the idea that providing knowledge about the causes of ill health and the choices available will result in changes to attitudes and practices, to minimize the disease burden.^1^ Several studies around the world have documented that exposure to daily passive smoking at home (usually from a partner) is an important risk factor for adverse health outcomes among mothers and their children*.*^2^^,^^3^^,^^4^^,^^5^ Therefore, to prevent the burden of diseases relating to secondhand smoking (SHS) and to reduce tobacco consumption, it is important to improve women’s knowledge and awareness regarding the risks of SHS.

A number of studies have assessed knowledge/attitudes regarding the risks of SHS in different population subgroups such as college and university students,^6^^,^^7^ healthcare professionals^7^^,^^8^ and ethnic minorities.^9^ However, only very few studies have targetedwomen,^10^^,^^12^ the group that bears the maximum brunt of SHS at home.

Previous studies that focused on women were conducted on restricted subgroups such as pregnant or working women with a higher educational profile. Since these studies ignored non-pregnant and uneducated or lower-educated women, they may have overestimated the magnitude of knowledge of the risks of SHS and attitudes towards these risks and may not reflect the real general situation among women. In addition, no studies have been conducted in Bangladesh to investigate knowledge and attitudes regarding the risks of SHS among women at home, despite the fact that nearly 40% of children^13^ and 53.5% of women^14^ are exposed to SHS at home in this country.

Up to now, women’s knowledge and attitudes regarding the risks of SHS and their relationship to exposure to SHS at home have not been thoroughly investigated. There are several possible ways in which women’s knowledge and attitudes regarding the dangers of SHS can influence exposure to SHS at home. It is believed that if women have relevant knowledge regarding the risks of SHS, they will be able to defend themselves against smoking perpetrated by men in these women’s homes by imposing various degrees of restrictions on home smoking such as insisting that these men should do this privately or when out of the house. If women have proper knowledge and non-dismissive attitudes about the adverse health effects of exposure to SHS, they can also combat smoking perpetrated by men such as their husbands in their homes by convincing them that as good fathers, they have the responsibility to protect the family from the hazardous effects on health caused by exposure to SHS and therefore should not smoke at home.

Furthermore, knowledge and attitudes regarding the risks of SHS need to be examined in relation to different socioeconomic strata in low-resource settings like Bangladesh, where rapid industrialization and urbanization over recent decades have increased socioeconomic inequality.^15^ In Bangladesh, it has been shown that there is a significant gradient in smoking prevalence across different socioeconomic groups,^14^ such that the lowest socioeconomic group has the highest prevalence rates of smoking.^14^

Although epidemiological studies have found significant relationships between socioeconomic status (SES) and smoking behavior, studies on differences in knowledge/attitudes regarding the risks of SHS according to SES are scarce. Moreover, the relationship between SES and knowledge and attitudes regarding the risks of SHS remains unknown among Bangladeshi women in general. Women of low SES may be at a distinct disadvantage because of higher levels of smoking among their partners.^15^ These women’s lack of resources can restrict their development, educational opportunities, access to healthcare and decision-making autonomy,^16^ thus creating a favorable setting for lack of knowledge and dismissive attitudes regarding this subject.

## OBJECTIVE

We aimed to go further into this important field of inquiry by addressing (1) whether SES and exposure to SHS were independently associated with knowledge and attitudes regarding the risks of SHS; and (2) whether women in the group with low SES and exposure to SHS were uniquely disadvantaged in terms of deficient knowledge and more dismissive attitudes towards the risks of SHS.

## METHODS

### Design and population

This study had a cross-sectional design and was conducted in the Rajshahi district of Bangladesh, covering both rural and urban areas. Households were surveyed and female parents with one child younger than five years were selected in each of the households thus identified. We picked out the households that had at least one adult smoker and a non-smoking mother. Administratively, the urban area in the Rajshahi district is split into wards and the rural region is split into union parishads (UPs). In Bangladesh, wards are elective units of cities or towns and UPs are the smallest rural administrative and local government units.

The sample size was calculated using the following formula: n=z^2^xpxq/m^2^; where “n” is the number of subjects required; z is the 95% confidence level (standard value of 1.96); P = 53.5% (assuming exposure to SHS among women at home in Bangladesh);^14^ q= 1 - P = 46.5%; and m = precision rate (value of 0.06). Thus, n = (1.96^2^ x 0.535 x 0.465) / 0.06^2^ = 265. Considering a design effect of 2, the minimum sample size became 530. We further increased our sample size to 541 to increase the power of the study. Thesample was then further allocated to equal ratios in the rural area (n=272) and urban area (n = 269)

A two-stage sampling approach was taken for selecting the households in the urban and rural areas of the Rajshahi district. In the first stage, out of 30 wards in the urban area of the Rajshahi district, two wards were randomly selected; and out of 70 UPs in the rural area, two were randomly selected. Since our target populations were not well identified or accessible, we therefore used the snowball sampling technique in the second phase. In this, we focused on one or two key individuals, who, we believed, knew about the field of study that we were investigating.

### Questionnaire

The survey questionnaire was developed from the World Health Organization’s Global Youth Tobacco Survey (GYTS),^17^ in combination with questions retrieved from a demographic and health survey (DHS) that was conducted in Bangladesh.^18^ Additionalquestions on knowledge and attitudes regarding SHS were developed by the project staff through reviewing relevant measures and related papers,^11^^,^^12^^,^^16^^,^^19^ and these were tested extensively in the field. The questionnaires were outlined in English and then translated into Bangla, the national language of Bangladesh. Thetranslation was judged by experts and volunteers. The content validity of the initial questionnaire was evaluated via a pilot test. The questionnaire was firstly pre-tested on 10% of the entire sample (n = 54) that were not selected for the survey. After correction of ambiguities that were identified in the questionnaires, the survey was administered in May and July 2017 (data not shown).

We also examined the reliability, internal consistency and reproducibility of the questionnaire. With regard to internal consistency, the homogeneity of the questions on knowledge and attitudes was evaluated using Cronbach’s α coefficient. The Cronbach’s α results were 0.77 and 0.72 for the knowledge and attitude instruments. Regarding reproducibility, the two sets of answers from the patients in the test-retest group were examined using the intraclass correlation coefficient. A coefficient of 0.70 or higher was considered to be evidence of satisfactory test-retest reliability.^20^

### Interviews and sources of potential bias

Trained and experienced field researchers conducted all household visits. There were seven interviewer teams, and each team comprised two interviewers. All of them received three days oftraining and two days of virtual sessions on the substanceof the questionnaire, techniques to elicit more information and strategies for obtaining complete and dependable information. Forclarification of the purpose of the research, an operational manual for interviewers and supervisors was provided two days before the training started, to ensure that they understood their duties and responsibilities.

Data quality standards were maintained through various actions. Since the sample only comprised women, we therefore only enrolled female interviewers. There were two quality control teams, and each team comprised one male and one female staff person. They were sent into the field to visit the interviewing teams throughout the data collection period. They observed one household and one individual interview conducted by each interviewer team and spot-checked the completed questionnaires. Theteams also revisited half of the households seen by each study teamand checked whether the households selected had been visited, andwhether the eligible respondents had been properly named and questioned. Debriefing sessions were held between the fieldworkers’ tours of duty to discuss any problems encountered in the field, provide clarifications and deal with administrative matters.

### Measurements

First, we registered several sociodemographic and health-related variables: respondent’s age, woman’s education, husband’s education, number of people in household, place of residence, woman’s decision-making autonomy, religion, marital status and respondent’s occupation.

The degree of knowledge and dismissiveness of attitudes towards the risks of SHS were the outcomes of interest in this study. Thequestionnaire on knowledge that was used in this study, which was modified from the GYTS mentioned above, consisted of five questions regarding: (1) awareness of the adverse effects on the health of children and adults caused by SHS; (2) awareness that children are more vulnerable to SHS than are adults; (3) awareness that SHS causes reproductive health problems among women; (4) awareness that smoking is prohibited in public places in Bangladesh; and (5) awareness that no legislation making homes smoke-free zones exists in Bangladesh. The questions on the women’s knowledge were chosen based on inspection of relevant standards and related papers.^11^^,^^12^^,^^16^^,^^19^ If the respondents gave positive responses to questions 1 to 4, scores of 1 point were given; otherwise, the score was “0”. For question number 5, if the respondents gave a negative response, a score of 1 point was given. This yielded a total possible score of 5 points.

We used six statements to determine whether the women had dismissive attitudes towards the risks of SHS: (1) smoking should be totally banned in all public places; (2) smoking should not be allowed at home; (3) I have the right to require other people not to smoke in my presence; (4) presence of SHS encourages young people to begin to smoke; (5) I believe that allowing SHS at home discourages smokers from quitting; and (6) It is difficult to adopt a no-smoking policy at home. If the respondents agreed with statements 1 to 5, they scored 1 point for each of these. Ifthe respondents disagreed with statement 6, they scored 1 point. Otherwise,the score was “0”.

To obtain information for measuring the women’s decision-making autonomy, the following questions were asked: 1)who decides how the household’s income will be used? 2) who hasthe final say in making large household purchases? 3) who has the final say about making household purchases for daily needs? 4)who hasthe final say regarding the woman’s own healthcare? 5)whohas the final say regarding child healthcare? And 6) who has the final say on visits to family or relatives? For each of these questions, the responses were coded as: 1) respondent; 2) respondent and husband/partner jointly; 3) respondent and someone else; 4) husband/partner; or 5) someone else in the house. To assess the respondent’s autonomy from these responses, binary variables were created for each of the questions. Responses 1, 2, and 3 were merged into a single category of having decision-making power; and responses 4 and 5 were merged into a single category indicating no decision-making power. From this, the decision-making power was ranked in terciles as low, medium or high.

### Outcomes and statistical analysis

Exposure to SHS and socioeconomic status were the exposures of interest in this study. The women’s self-reports were used to assess their exposure to SHS. They were asked, “Is smoking prohibited at home?” Those who responded “no” were considered to be exposed to SHS at home. We focused on standard of living (hereinafter referred to as wealth) as a measurement of SES. A wealth index was constructed from data on household assets, including ownership of durable goods (such as radio, television, mobile phone, landline phone, freezer, almirah/wardrobe, table, chair, watch, electric fan and DVD/VCR player), ownership of means of transportation (such as bicycle, motorcycle/scooter/tempo, car/truck, rickshaw/van or cart), ownership of agricultural land (such as homestead or other agricultural land) and access to electricity.

As a rule of thumb in constructing this wealth index, variables with prevalences below 3-5% (such as sources of drinking water, sources of toilet facilities, access to electricity, car/truck, landline phone and cart)^21^ were excluded from the analysis. Each asset was assigned a weight (factor score) that was generated through principle component analysis, and the resulting asset scores were standardized in relation to a standard normal distribution with a mean of zero and a standard deviation of one. Each household was then assigned a score for each asset, and the scores were summed per household. The sample was then divided into terciles; each tercile was designated a rank, from one (poor) to three (rich), and individuals were ranked according to the total score of the household in which they lived.

We provided descriptive statistics for sociodemographic data, exposure to SHS and knowledge and attitude-related characteristics in our sample. Differences in knowledge and attitude between the exposed and non-exposed groups were assessed by means of cross-tabulation. Because the outcomes measured were ordinal, adjusted ordered logistic regressions were used. Parallelline tests confirmed that the proportional odds assumption was not violated. We included the following independent variables as potential confounders for the events in the logistic model: covariates of respondent’s age, woman’s education, husband’s education, number of people in household, place of residence, woman’s decision-making autonomy, religious belief and marital status.

All the covariates were entered simultaneously into the multiple regression models. The significance level for all analyses was set at P < 0.05. To ascertain whether the women who were in the group with low SES and exposure to SHS uniquely presented lower levels of knowledge about exposure to SHS and more dismissive attitudes towards the risks of SHS, we conducted ordinal logistic regression analyses to examine the adjusted association between SHS and knowledge and attitudes regarding exposure to SHS after stratification according to wealth level. We estimated odds ratios (ORs) to assess the strength of the associations and used 95% confidence intervals (CIs) for significance testing.

Indices of knowledge and attitudes were constructed using the sum of weighted binary input variables, and maximum and minimum values were chosen for each underlying indicator. Theperformance of each indicator was expressed through a unit-free index with values between 0 and 1 (which allows different indices to be added together), in accordance with the construction method of the Human Development Index,^22^ as follows:

Dimension index = (actual value-minimum value)/(maximum value-minimum value)

The scores obtained for each of the indices were then recoded as terciles, with categories labeled low, middle and high knowledge and attitudes. The Statistical Package for the Social Sciences software (Version 22.0, Chicago, IL, USA) was used for performing all statistical analyses.

### Ethical considerations

This study protocol was reviewed and approved by the ethics committee of the Institute of Biological Sciences, University of Rajshahi, Bangladesh (approval number 74/320/IAMEBBC/BSc, dated February 22, 2017). Prior to the survey, potential participants were informed about the study, invited to participate and informed of their right to decline to take part. All medical waste materials used for this study were disposed of safely.

## RESULTS

### Descriptive statistics

Table 1 shows self-reported exposure to SHS among the women according to their sociodemographic characteristics. A total of 541 women were included in the study. The prevalence of self-reported exposure to SHS was found to be 49.0%.

**Table 1.** Self-reported exposure to secondhand smoking among married women, according to sociodemographic characteristics (n = 541)

Characteristics	n (%)	Self-reported exposure to secondhand smoking
Age, years		
15-22	208 (38.4)	110 (41.5)
23-26	155 (28.7)	75 (28.3)
27-45	178 (32.9)	80 (30.2)
P-value		0.294
Education		
No education	37 (6.8)	24 (9.1)
Primary	108 (20.0)	52 (19.6)
Secondary	324 (59.9)	166 (62.6)
Higher secondary and above	72 (13.3	23 (8.7)
P-value		0.005
Husband’s education		
No education	98 (18.1)	59 (22.3)
Primary	167 (30.9)	87 (32.8)
Secondary	199 (36.8)	88 (33.2)
Higher secondary and above	77 (14.2)	31 (11.7)
P-value		0.021
Marital status		
Divorce/separated/widowed	12 (2.2)	5 (1.9)
Currently married	529 (97.8)	260 (98.1)
P-value		0.608
Parity		
1	242 (44.7)	120 (45.3)
2	207 (38.3)	102 (38.5)
3+	92 (17.0)	43 (16.2)
P-value		0.892
Decision-making autonomy*		
Low	186 (34.4)	124 (46.8)
Medium	89 (16.5)	57 (21.5)
High	266 (49.2)	84 (31.7)
P-value		< 0.001
Religion		
Non-Muslim	14 (2.6)	9 (3.4)
Muslim	527 (97.4)	256 (96.6)
P-value		0.246
Occupation		
Employed	489 (90.4)	12 (4.5)
Household work	37 (6.8)	247 (93.2)
Unemployed/student	15 (2.8)	6 (2.3)
P-value		0.082
Place of residence		
Rural	272 (50.3)	183 (69.1)
Urban	269 (49.7	82 (30.9)
P-value		< 0.001
Number of people in household		
3-4	181 (33.5)	80 (30.2)
5-6	205 (37.9)	94 (35.5)
7+	155 (28.7)	91 (34.3)
P-value		0.016
Socioeconomic status		
Poor	180 (33.3)	112 (42.3)
Middle	180 (33.3)	92 (34.7)
Rich	181 (33.5)	61 (23.0)
P-value		< 0.001
Prevalence		265 (49.0)

*Aspects of family decision-making that the woman participated in, alone or jointly. BMI = body mass index (defined as weight in kilograms divided by the square of height in meters); BMI categories were underweight (< 18.5), normal (18.5-24.9), or overweight/obese (≥ 25).

Table 2shows the differences in knowledge regarding SHS between the exposed and non- exposed groups. The non-exposed group was significantly more knowledgeable in relation to all the indicators of knowledge, in comparison with the exposed group. Regarding knowledge grading scores, the women in the non-exposed group had significantly higher scores for levels of knowledge (P < 0.001) than those of their counterparts (32.2% versus 7.5%). However, out of the total sample, only one fifth of the respondents (20.1%) had higher levels of knowledge regarding SHS.

**Table 2.** Knowledge of and attitudes towards the risks of secondhand smoking (SHS) among the women according to whether they were in the exposed or non-exposed group (n = 541)

Variables	n (%)			P-value
	Exposed	Non-exposed	Total	
Knowledge regarding risks of SHS				
SHS has adverse health effects on children and adult	129 (48.7)	210 (76.1)	339 (62.7)	< 0.001
Children are more vulnerable to SHS than are adults	109 (41.1)	202 (73.2)	311 (57.5)	< 0.001
SHS causes reproductive health problems among women	125 (47.2)	161 (58.3)	286 (52.9)	0.009
Smoking is prohibited in public places in Bangladesh	128 (48.3)	175 (63.4)	303 (56.0)	< 0.001
There are no laws making homes smoke-free in Bangladesh	81 (30.6)	183 (66.3)	264 (48.8)	< 0.001
Knowledge grading				
Low	115 (43.4)	55 (19.9)	170 (31.4)	< 0.001
Medium	130 (49.1)	132 (47.8)	262 (48.4)	
High	20 (7.5)	89 (32.2)	109 (20.2)	
Attitudes towards the risks of SHS				
Smoking should be completely banned in all public places	165 (62.3)	164 (59.4)	329 (60.8)	0.498
Smoking should be prohibited at home	95 (35.8)	263 (95.3)	358 (66.2)	< 0.001
I have the right to ask other people not to smoke in my presence	145 (54.7)	183 (66.3)	328 (60.6)	0.006
It is difficult to adopt a no-smoking policy at home	152 (57.4)	89 (32.2)	241 (44.5)	< 0.001
Presence of SHS encourages young people to begin to smoke	183 (69.1)	237 (85.9)	420 (77.6)	< 0.001
I believe that allowing SHS at home discourages smokers from quitting	92 (34.7)	263 (95.3)	355 (65.6)	< 0.001
Attitude grading				
Low	142 (53.6)	72 (26.1)	214 (39.6)	< 0.001
Medium	73 (27.5)	52 (18.8)	125 (23.1)	
High	50 (18.9)	152 (55.1)	202 (37.3)	

Table 2 also shows that there were differences in attitudes towards the risks of SHS between the exposed and non-exposed groups. The non-exposed group had significantly more non-dismissive attitudes towards the risks of SHS, in comparison with the exposed group, except in relation to the attitude of finding it difficult to prohibit smoking at home. Regarding attitude grading scores, the women in the non-exposed group had significantly higher scores for dismissive attitudes (P < 0.001), compared with their counterparts (55.1% versus 18.9%). Out of the total sample, 37.3% of the respondents had higher levels of dismissive attitudes towards the risks of SHS.

The poor women (8.3%) had the lowest scores for levels of knowledge, in comparison with the medium-wealth (17.2%) and rich women (34.8%), while the non-exposed group in all the three socioeconomic classes had higher scores for levels of knowledge than those of the exposed group. The poor women (18.9%) also had the lowest scores for dismissive attitudes, in comparison with the middle-wealth (33.9%) and rich women (59.1%), while the non-exposed group in all the three socioeconomic class had higher scores for dismissive attitudes than those of the exposed group. **Supplementary**Figures 1and2show the distribution of knowledge and attitude grading according to the exposed and non-exposed groups after stratification in terms of SES.

**Figure 1.**
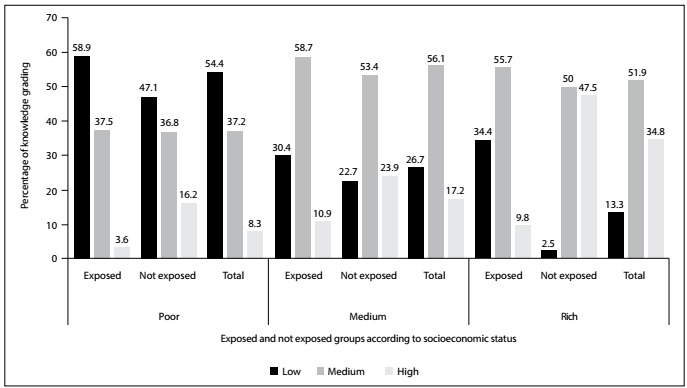
Distribution of knowledge grading according to exposure or non-exposure to secondhand smoking, after stratification according to socioeconomic status.

**Figure 2.**
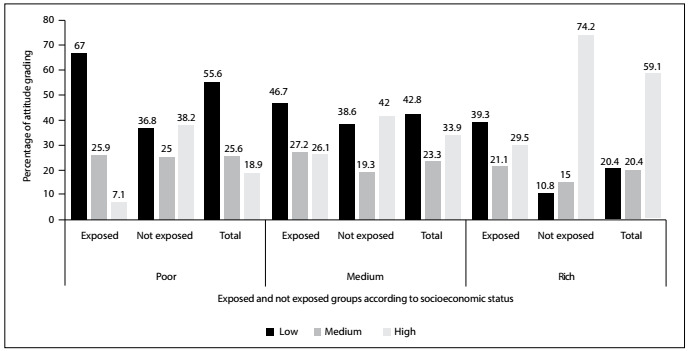
Distribution of attitude grading according to exposure or non-exposure to secondhand smoking, after stratification according to socioeconomic status.

Table 3 shows the adjusted ordinal logistic regression model for the association between knowledge grading and self-reported exposure to SHS, SES and other sociodemographic factors. In the adjusted model, the respondents who reached the secondary level of education (adjusted odd ratio, AOR = 6.06; 95% confidence interval, CI = 2.56-14.38) or who reached the higher secondary level or above (AOR = 8.46; 95% CI = 2.96-24.13) were more likely to have high knowledge scores than to have knowledge in the combined medium and low categories, in comparison with respondents who had not had any education.

**Table 3.** Odds ratios for the associations between knowledge/attitude grading and low socioeconomic status (SES), secondhand smoking (SHS) and other sociodemographic factors (n = 541)

Variables	Adjusted odds ratio (95% CI)	
	Knowledge of the risks of SHS	Non-dismissive attitudes towards the risks of SHS
Age, years		
15-22	1.00	1.00
23-26	0.99 (0.62-1.58)	1.79 (1.08-2.95)^*c*^
27-45	0.69 (0.41-1.17)	0.92 (0.52-1.62)
Education		
No education	1.00	1.00
Primary	2.40 (0.99-5.78)	1.68 (0.63-4.48)
Secondary	6.06 (2.56-14.38)^*a*^	3.05 (1.17-7.91)^*c*^
Higher secondary and above	8.46 (2.96-24.13)^*a*^	8.03 (2.54-25.35)^*a*^
Husband’s education		
No education	1.00	1.00
Primary	0.92 (0.53-1.59)	0.86 (0.48-1.54)
Secondary	0.87 (0.50-1.53)	0.65 (0.36-1.18)
Higher secondary and above	0.70 (0.33-1.48)	0.61 (0.27-1.36)
Marital status		
Divorce/separated/widowed	1.00	1.00
Currently married	0.88 (0.26-3.00)	0.87 (0.22-3.37)
Parity		
1	1.00	1.00
2	1.35 (0.86-2.11)	0.56 (0.35-0.91)^*c*^
3+	1.46 (0.75-2.85)	0.57 (0.27-1.16)
Decision-making autonomy		
Low	1.00	1.00
Medium	2.18 (1.26-3.78)^*b*^	1.70 (0.94-3.07)
High	6.33 (3.98-10.08)^*a*^	2.63 (1.60-4.32)^*a*^
Religion		
Non-Muslim	1.00	1.00
Muslim	1.15 (0.35-3.80)	0.99 (0.31-3.19)
Occupation		
Employed	1.00	1.00
Household work	1.59 (0.77-3.30)	0.55 (0.25-1.23)
Unemployed/student	1.69 (0.46-6.19)	0.43 (0.10-1.77)
Place of residence		
Rural	1.00	1.00
Urban	2.45 (1.61-3.87)^*a*^	1.62 (1.03-2.55)^*c*^
No. of people in household		
3-4	1.00	1.00
5-6	0.96 (0.60-1.52)	1.38 (0.83-2.29)
7+	0.88 (0.54-1.41)	1.59 (0.96-2.64)
Knowledge of the risks of SHS		
Low	---	1.00
Medium		5.99 (3.63-9.90)^*a*^
High		26.09 (12.68-53.68)^*a*^
SHS		
Not exposed	1.00	1.00
Exposed	0.65 (0.44-0.98)^*c*^	0.57 (0.38-0.87)^*b*^
Socioeconomic status		
Poor	1.00	1.00
Middle	2.82 (1.78-4.47)^*a*^	0.93 (0.57-1.51)
Rich	4.55 (2.73-7.60)^*a*^	1.88 (1.08-3.28)^*c*^

CI = confidence interval. a, b and c indicate P < 0.05, P < 0.01 and P < 0.001.

Women with medium-level decision-making autonomy (AOR= 2.18; 95% CI = 1.26-3.78) or high-level decision-making autonomy (AOR = 6.33; 95% CI = 3.98-10.08), in comparison with low decision-making autonomy, were more likely to be associated with high knowledge scores than with the combined medium and low categories. Women belonging to the medium SES group (AOR = 2.82; 95% CI = 1.78-4.47) or rich SES group (AOR = 4.55; 95% CI= 2.73-7.60), in comparison with the poor SES group, were more likely to have high knowledge scores than to be in the combined medium and low categories. Likewise, the odds of being in the combined middle and high-knowledge categories rather than in the low-knowledge category were 6.06, 8.46, 2.18, 6.33, 2.82 and 4.55 times greater for respondents who reached secondary-level education or higher secondary and above, those with medium or high autonomy and those belonging to the middle or rich SES groups. In addition, respondents in the exposed group had 0.65 times lower odds of having higher knowledge scores.

Table 3 also shows the adjusted ordinal logistic regression model for the association between attitude grading and self-reported exposure to SHS, SES and other sociodemographic factors. In the adjusted model, respondents aged 23-26 years (AOR = 1.79; 95% CI = 1.08-2.95), versus 15-22 years, were more likely to be associated with high scores for dismissive attitudes than with the combined medium and low categories. Respondents who reached secondary-level education (AOR = 3.05; 95% CI = 1.17-7.91) or higher secondary and above (AOR = 8.03; 95% CI = 2.54-25.35), rather than having no education, were more likely to be associated with high scores for non-dismissive attitudes than with the combined medium and low categories.

Women with high decision-making autonomy (AOR = 2.63; 95% CI = 1.60-4.32), rather than low decision-making autonomy, were more likely to be associated with high scores for non-dismissive attitudes than with the combined medium and low categories. Likewise, the odds of being in the combined middle and high-score categories for dismissive attitudes, rather than the low-score category were 1.79, 3.05, 8.03 and 2.63 times greater for respondents aged 23-26 years, women with secondary-level education or higher secondary and above and women with high autonomy. Women living in rural areas, belonging to the rich wealth bands and having medium or high levels of knowledge grading scores presented higher odds of having higher scores for dismissive attitudes. In addition, women with parity of two and women belonging to the exposed group had 0.56 and 0.57 times lower odds of having higher scores for dismissive attitudes.

Table 4 shows the adjusted ordinal logistic regression model for the association between knowledge grading and the exposure or non-exposure group after stratification according to SES. Womenbelonging to the low SES group who were exposed to SHS were less likely to have high knowledge scores (AOR = 0.48; 95% CI= 0.25-0.90) than were the women belonging to the low SES group who were not exposed to SHS. Rich women who were exposed to SHS were less likely to have high knowledge scores (AOR = 0.20; 95% CI = 0.07-0.52) than were the women belonging to the rich wealth bands who were not exposed to SHS.

**Table 4.** Odds ratios for associations between knowledge/attitude grading and belonging to the exposed or non-exposed group to secondhand smoking (SHS), stratified according to socioeconomic status (n = 541)

Variable	Poor	Middle	Rich
	Adjusted odds ratio (95% confidence interval)		
Knowledge			
SHS			
Not exposed	1.00	1.00	1.00
Exposed	0.48 (0.25-0.90)^*c*^	0.80 (0.40-1.57)	0.20 (0.07-0.52)^*b*^
Attitude			
SHS			
Not exposed	1.00	1.00	1.00
Exposed	0.26 (0.12-0.58)^*b*^	0.99 (0.51-1.92)	0.36 (0.15-0.87)^*c*^

^1,2,3^Models were adjusted according to age, education, husband’s education, marital status, parity, decision-making autonomy, religion, occupation, place of residence, number of people in household and exposure to SHS. a, b and c indicate P < 0.001, P < 0.01 and P < 0.05.

Table 4 also shows adjusted ordinal logistic regression model for the association between attitude grading and the exposed and non-exposed groups after stratification according to SES. Womenbelonging to the low SES group who were exposed to SHS were less likely to have high scores for dismissive attitudes (AOR = 0.26; 95% CI = 0.12-0.58) than were the women belonging to the low SES group who were not exposed to SHS. Rich women who were exposed to SHS were less likely to have high scores for dismissive attitudes (AOR = 0.36; 95% CI = 0.15-0.87) than were the women belonging to the rich wealth bands who were not exposed to SHS.

## DISCUSSION

To the best of our knowledge, this is the first study to assess the relationships between exposure to SHS, SES and knowledge of and attitudes towards the risks of SHS among married Bangladeshi women of reproductive age. We found that the prevalence of exposure to SHS at home among our sample was 49.0%. In comparison, a study conducted in Bangladesh among the adult population found a prevalence of 43.0%.^14^

The current levels of exposure to SHS at home among these married women are worrisome and constitute a matter for concern for public health researchers and practitioners. The findings likewise indicate that although the bulk of these women had knowledge regarding various indicators for exposure of SHS, their overall knowledge scores are lower (20.2%). Regarding attitude grading scores, only 37.3% of the respondents had more non-dismissive attitudes towards the risks of SHS.

This survey showed that there was a statistically significant correlation between higher knowledge and high scores for non-dismissive attitudes, in relation to exposure to SHS. Thisresult was expected, since the respondents in the exposed group had low knowledge and low levels of non-dismissive attitudes and were therefore more likely to be exposed to SHS. Recently, several studies showed that poor awareness and knowledge regarding the risks of SHS were barriers hindering progress.^23^^,^^24^^,^^25^ Conversely,good awareness and knowledge of the risks acted as a motivator.^26^ Whenwomen were aware that exposure “presented a risk,”this motivated them to make behavioral changes regarding smoking at home.^23^Thesefindings therefore indicate that urgent effective interventions are needed in order to raise the level of knowledge and establish a good non-dismissive attitude towards avoidance of exposure to SHS among women.

Socioeconomic status is an important determinant of health and wellbeing because it influences people’s attitudes, experiences and exposure to several health risk factors.^27^ Indeed, several studies have shown that low SES is related to presence of a variety of chronic diseases and to all-cause mortality because of these individuals’ lack of knowledge.^28^^,^^29^ In line with these findings, our study also showed that participants in the low SES group were less likely to have high levels of knowledge of and more likely to have dismissive attitudes towards the risks of SHS, compared with individuals in the high SES group.

Consistent with the findings from previous studies in Bangladesh,^14^^,^^30^ we also found high levels of knowledge of and non-dismissive attitudes towards the risks of SHS among the respondents who had reached at least the primary level of education. To maintain this high level of knowledge among thewomen, as well as among the rest of the adult population, the existing promotional campaigns towards tobacco control need to be continued on a regular basis. Graphic warning labels could be successful in reaching illiterate populations*.* Because there are differences in knowledge according to educational level, targeted campaigns with customized messages should be designed to reach illiterate populations.

Our findings also showed that rural respondents were less likely to have higher levels of knowledge and higher grading scores for non-dismissive attitudes. One possible explanation for this result is that in rural areas, the population may lack information and knowledge about passive smoking and may have a lower educational level than that of urban women. Another possible explanation is that, compared with rural areas, urban areas often participate in anti-smoking campaigns and receive tobacco control education,^31^ thereby leading to greater knowledge and more non-dismissive attitudes towards the risks of SHS.

We also found that middle-aged mothers (23-26 years) and mothers with high decision-making power were more likely to have higher knowledge and more non-dismissive attitudes than were their counterparts. The possible explanation for this is that middle-aged women and women with higher autonomy have usually reached higher education levels.^32^ Additional analyses were run to support this hypothesis, and we found that that the middle-aged women and women with higher autonomy did indeed have higher levels of education. There was also a significant positive correlation between knowledge of and non-dismissive attitudes towards the risks of SHS. Hence, it seems that high levels of knowledge could lead to good levels of non-dismissive attitudes towards the risks of SHS. This result was similar to the findings from a previous study, in which it was found that knowledge pertaining to smoking was predictive of having non-dismissive attitudes towards the risks of smoking, and that this contributed towards effective tobacco control.^33^

Our findings also demonstrated that in relation to higher levels of knowledge and higher scores for non-dismissive attitudes, women who were exposed to SHS and belonged to the poor SES group were not uniquely disadvantaged. Therefore, it is the exposure to SHS per se that disadvantages women, whereas belonging to the low SES group does not uniquely disadvantage women who are exposed to SHS. The importance of this finding needs to be underscored. When exposure to SHS adversely impacts women’s knowledge and attitudes, it does so whether the woman has low SES or not. Because of the lack of high levels of knowledge and non-dismissive attitudes, the negative effect of exposure to SHS extends across all socioeconomic backgrounds and is not limited to women who belong either to the low or to the high SES group.

Some limitations need to be considered in explaining our findings. Foremost, the cross-sectional design of the study limits causal inferences about determinations. Secondly, the current paper focused on household exposure to SHS and did not identify other sources of vulnerability such as in workplaces or outdoors. Therefore, our findings cannot be generalized to exposure to SHS outside of the home.

Thirdly, there may have been the possibility of underreporting of self-reported exposure to SHS. To decrease this underreporting, the following strategies were used: in-person interviews were used rather than a self-administered questionnaire; the questions were behaviorally specific; the women were given several opportunities to reveal their level of knowledge and their attitudes towards the risks of SHS at home within the same interview; and efforts were made to create an atmosphere of trust.

Fourthly, there may have been the possibility of confounding bias. Nevertheless, the confounders that were adjusted for in the present investigation were the factors that are most usually found to be interrelated with exposure to SHS, and to knowledge and attitudes relating to this within the contexts studied. Moreover,the socioeconomic index that was used in the present investigation reflected all the relevant factors associated with the situation of poverty.

Lastly, since the sampling frame was not known, and the sample was not chosen randomly, it is unlikely to have been representative of the population that was studied. However,the findings from this study might be relevant to other areas in Bangladesh and to neighboring low-income countries. Thestudy areas manifested the typical features of rural and urban Bangladesh. Thefindings were generally consistent with those from other culturally and ethnically different study populations in Bangladesh.

## CONCLUSIONS

Exposure to SHS and low SES were independently associated with deficient levels of knowledge and higher scores for dismissive attitudes regarding the risks of SHS. The findings also revealed that because of the lack of high levels of knowledge and because of the high scores for dismissive attitudes regarding the risks of SHS, the negative effects of exposure to SHS extended across all socioeconomic backgrounds and were not limited to women who belonged either in the low or in the high SES group. Future research is needed to understand the causal structures between the exposures and desired outcomes.
